# BEING WOMEN AND BEING DEMENTIA CAREGIVERS: SELF-CONCEPTS IN THE MAKING OF VIETNAMESE FEMALE CAREGIVERS

**DOI:** 10.1093/geroni/igz038.3345

**Published:** 2019-11-08

**Authors:** Trang T Nguyen

**Affiliations:** Center for Studies of Displaced Populations, Tulane University, New Orleans, Louisiana, United States

## Abstract

In Vietnam, the majority of dementia caregivers are women. They play multiple social roles, and confront role conflicts and caregiving burdens with insufficient social supports. Dementia caregiving alters their self-concepts, or who they think they are. This paper aims to explore self-concepts of Vietnamese female caregivers of older relatives with Alzheimer’s Disease (AD). In total, 21 face-to-face, semi-structured interviews, including six follow-up interviews, with 13 Vietnamese female caregivers of older patients with AD were conducted. These 13 caregivers were from 44 to 71 years old, mostly spouses of the patients with AD (n = 8), and retired (n = 9). Thematic coding procedure and the program MaxQDA12 were used for data analysis. Results show that the self-concepts of female caregivers in dementia care were complex, contextualized, and manifested in different aspects. First, self-concepts of these female caregivers were the outcome of the interactions between the guided-self and the performed-self. Their guided-self was the self that their social norms and cultural traditions told them about who they should be, while their performed-self was the self they demonstrated to the outside world. The mismatch between these two types of self caused distress among caregivers. Second, caregivers’ self-concept was the combination of the three key types of the self: the moral-self (a filial daughter or a responsible wife); the feminine-self (a patient and graceful women); and the worthy-self (a devoted and helpful caregiver). Understanding Vietnamese women’s self-concepts associated with their sociocultural context will better inform the development of support programs for them.

